# Bifurcation analysis of the dynamics of interacting subnetworks of a spiking network

**DOI:** 10.1038/s41598-019-47190-9

**Published:** 2019-08-06

**Authors:** Fereshteh Lagzi, Fatihcan M. Atay, Stefan Rotter

**Affiliations:** 1 Bernstein Center Freiburg, Freiburg, Germany; 2 Faculty of Biology, University of Freiburg, Freiburg, Germany; 3 Department of Mathematics, Bilkent University, Ankara, Turkey

**Keywords:** Network models, Applied mathematics

## Abstract

We analyze the collective dynamics of hierarchically structured networks of densely connected spiking neurons. These networks of sub-networks may represent interactions between cell assemblies or different nuclei in the brain. The dynamical activity pattern that results from these interactions depends on the strength of synaptic coupling between them. Importantly, the overall dynamics of a brain region in the absence of external input, so called ongoing brain activity, has been attributed to the dynamics of such interactions. In our study, two different network scenarios are considered: a system with one inhibitory and two excitatory subnetworks, and a network representation with three inhibitory subnetworks. To study the effect of synaptic strength on the global dynamics of the network, two parameters for relative couplings between these subnetworks are considered. For each case, a bifurcation analysis is performed and the results have been compared to large-scale network simulations. Our analysis shows that Generalized Lotka-Volterra (GLV) equations, well-known in predator-prey studies, yield a meaningful population-level description for the collective behavior of spiking neuronal interaction, which have a hierarchical structure. In particular, we observed a striking equivalence between the bifurcation diagrams of spiking neuronal networks and their corresponding GLV equations. This study gives new insight on the behavior of neuronal assemblies, and can potentially suggest new mechanisms for altering the dynamical patterns of spiking networks based on changing the synaptic strength between some groups of neurons.

## Introduction

Networks of pulse-coupled units that operate with a threshold mechanism abound. Examples are forest fires, swarms of flashing fireflies, earthquakes and interacting spiking neurons. Representing the interactions in such systems by a directed graph, each node on the graph receives inputs from many other nodes. If the system input crosses a threshold, a pulse-like signal is emitted and transmitted to the neighboring nodes. The collective behavior of such systems is of particular interest for various reasons, for example, the ability to control the dynamics, or to predict certain events. For instance, in translational neuroscience, one needs to understand the circumstances under which runaway brain states emerge (like an epileptic seizure), or control pathological activity dynamics (like basal ganglia oscillations in Parkinson’s disease).

It is believed that the neocortex has a modular structure with modules that are similar in overall design and operation but different in cell types and connectivity^1^. This conceptualizes the brain as a hierarchical network of subnetworks that interact with each other, and as a consequence, build a functional brain. Moreover, due to spike timing synaptic plasticity, pre-synaptic neurons that fire together within a close time frame with post-synaptic neurons strengthen synaptic connections that eventually results in formation of cell assemblies^2^. Neurons in these assemblies can be connected via short or long range synapses. The interaction of these assemblies may shape an ongoing brain activity that exists even in the absence of external inputs, and may correlate with some internal cognitive states^3^. This also entails the formation of associative memory^4^ or synfire chains^5,6^. On the simulation level, it has been shown that a combination of plasticity mechanisms can lead to input-dependent formation of cell-assemblies^7–9^ which can play role in nonlinear computations.

In subcortical regions, specifically the basal ganglia are comprised of subnetworks (“nuclei”) whose neurons are often conceived as threshold units, sharing similar properties. The synaptic interaction between these subnetworks results in a host of different behaviors, which can often be correlated with either healthy or pathological state. The basal ganglia are connected to many other parts of the brain, including the neocortex and the thalamus. “Up-states” and “down-states”, as examples of dynamical states of neuronal networks, have previously been reported in striatum^10,11^. Cooperation or competition between the different subnetworks of the basal ganglia and certain cortical regions can be conceived as interacting subnetworks of excitatory and inhibitory neurons^12,13^ or only inhibitory neurons^14,15^. Diseases such as Parkinson’s or Huntington’s are related to dysfunctions in one or several of these subnetworks, or the interactions between them. Therefore, understanding the role each subnetwork plays in the dynamics of the large network of interconnected brain regions is important to eventually dissect the pathophysiology of these dysfunctional states. Devising novel therapies to alleviate or entirely abolish such pathologies depends on this insight.

To understand the nature of the global dynamics that emerges from these interactions, a theoretical framework is needed. There are different approaches to study the collective dynamics of networks, depending on the exact system in question. Mean-field methods and linear models are common routes taken in the study of the low-dimensional dynamics of spiking neuronal networks^16–19^. When nonlinearities are important, Wilson-Cowan equations^10^ are a prime candidate for such analysis.

In this manuscript, we suggest an alternative low-dimensional firing rate equation for populations of interacting spiking neurons with block-random connections. Originally, these equations were suggested to describe population dynamics in simple ecosystems, with only few interacting species, known as predator-prey dynamics^20,21^. The analogy between predator-prey systems and spiking neuronal networks is rooted in the competition for limited resources and survival. Prey increases its own population size by reproduction, and also increases the size of the predator population by feeding them. Predators, on the other hand, decrease the prey’s population size by feeding on them. A more subtle and more indirect type of interaction is the competition between different predator species that feed on the same type of prey, which results in decreased population sizes of the predators. Likewise, in spiking network dynamics, high activity of excitatory neurons results in a larger number of neurons that are susceptible of firing. This, in turn, results in a higher activity level of both excitatory and inhibitory populations. Inhibitory neurons, on the other hand, bring down the membrane potential levels of other neurons in the network, and as a consequence, reduce the probability of spike emission. Depending on the feedback structure, this could then lead to a reduction of global network activity. We will show that Generalized Lotka-Volterra (GLV) equations, can capture such collective dynamics quite well, and represent bifurcation diagrams that are validated by spiking network simulations. In other words, with numerical simulations of spiking networks with different coupling parameters, and different structures, we show that there is a qualitative equivalence between these population equations and the steady state behavior of networks of subnetworks of spiking neurons. It should be emphasized that, in this study, the focus is on the qualitative equivalence of GLV systems and spiking network dynamics, rather than the quantitative similarity of their fixed points and relaxation dynamics.

GLV equations have been suggested before as an abstract model of neuronal dynamics^22–26^, but a systematic generalization to “networks of interacting networks” of spiking neurons still needs to be devised (for more details see^27^). In^28^, the special case of solutions to the homogeneous GLV equations has been compared to the steady state behavior of point processes with excitatory and inhibitory couplings. In^26^, two different networks composed of three interacting populations were studied, and GLV equations were derived from a first order perturbation of the steady state solution of the associated Fokker-Planck equation. In that study, modulation of couplings “within” each subnetwork resulted in different global network dynamics, similar to the behavior of GLV equations. However, a systematic analysis of the effect of couplings “between” subnetworks, and therefore a topological dynamical equivalence between GLV systems and the collective dynamics of networks of interacting spiking subnetworks is still missing. GLV equations are known for their rich repertoire of nonlinear dynamics, such as stable pattern formation^29^, continuous attractors^30^, oscillations^31^, and chaos^23,32^. For the locust olfactory system, for example, it has been convincingly demonstrated that the transient dynamics as produced by GLV models can robustly encode sensory information^33,34^. It has been hypothesized that sensory representations are more robustly encoded in the transient dynamics of neurons, from baseline to steady state firing rate, as compared to the stationary activity of neurons in the olfactory system of locusts^35^. This behavior is also very well captured by GLV equations.

In our study, we compare the behavior of GLV systems to the behavior of networks of subnetworks of excitatory and inhibitory neurons, while coupling parameters between them are changed (bifurcation parameters). We perform a systematic bifurcation analysis of two different networks, each one composed of three subnetworks. An excitatory sub-population has a positive impact on its own activity (growth rate), and hence the analysis is different compared to most GLV studies so far. In other studies, the self-influence of the prey population is negative to reflect a limit on the amount of available resources. It will be shown that the stability of different fixed points of the GLV system depends on the coupling parameters. This dependence is similar to the stable behavioral changes of the corresponding spiking network in the normalized parameter space. Moreover, in a network with purely inhibitory interactions that follows the May-Leonard structure, oscillations of subnetworks emerge for some parameter regimes, as predicted by the bifurcation diagram of such a system. This suggests that the phase portraits of the collective dynamics of spiking networks are similar to those of the representative GLV systems, as the parameters of the system change. In other words, the topology of the flows in these networks and their GLV models, respectively, are similar. This similarity is not affected by the time scales of the two systems or the magnitude of the eigenvalues.

As a model for the collective dynamics of networks of spiking networks, GLV equations also provide us with useful intuition on how to control the dynamics of such networks, with the goal to reestablish a desired behavior, for example in diseased brains.

## Methods

We studied networks with leaky-integrate-and-fire neuron models with a membrane time constant of $\tau =20\,{\rm{ms}}$, and a reset potential of $10\,{\rm{mV}}$. Each neuron receives an additional external direct current input of $270\,{\rm{pA}}$. We decided to use a direct current (instead of the commonly used stationary Poisson spike trains) as an external input to each neuron because, for our study, any possible source of random symmetry breaking was to be avoided.

Each neuron *i*, in a network composed of *N* neurons, obeys the equation

1 $$\tau {\dot{v}}_{i}(t)=-\,{v}_{i}(t)+\tau \,\sum _{j=1}^{N}\,{J}_{ij}{S}_{j}(t)+{R}_{res}I$$

where *S*_*j*_(*t*) is the spike train of a pre-synaptic neuron *j*, which projects to the post-synaptic neuron *i*. A spike is a brief and stereotyped impulse that is generated when the membrane potential of the neuron reaches a threshold (here fixed at 20 mV). In the above equation, *v*_*i*_ is the membrane potential of neuron *i*, and *J*_*ij*_ is the amplitude of the post-synaptic potential (PSP) caused by spikes in neuron *j* impinging on neuron *i*. We considered PSPs of amplitude 0.09 mV for excitatory synapses. In Eq. (1), *I* is the external DC drive, and *R*_*res*_ is the input resistance of the neuron. In our simulations of network activity, to regard causality, a uniform synaptic transmission delay of ${t}_{d}=0.1\,{\rm{ms}}$ was used, coinciding with the step size *dt* for all network simulations.

All networks studied in this paper are randomly connected. The parameter $\varepsilon$ represents the probability of connection between any two neurons within an excitatory subnetwork. The number $p\varepsilon$ is the probability of connection between any two inhibitory neurons, or one excitatory and one inhibitory neuron. We chose *p* = 3 to bring the network close to a cortical column^36^.

### Network structure

We studied two different scenarios, each of which was characterized by three interacting subnetworks. First, we considered a network, composed of two excitatory subnetworks and one inhibitory subnetwork (EEI). Second, a network of three interacting inhibitory subnetworks was considered (III), which is a neuronal implementation of the May-Leonard system^37^.

For a coarse-grained description, we sum over the dynamics (Eq. (1)) of the membrane potentials of all individual neurons in each subnetwork.

2 $$\begin{array}{rcl}\tau {\dot{V}}_{m}(t) & = & -{V}_{m}(t)+\tau \,\sum _{n=1}^{3}\,{W}_{mn}{R}_{n}(t)+{N}_{m}{r}_{0}(t)\\  & = & -{V}_{m}+{L}_{m}(R).\end{array}$$

In this equation, *V*_*m*_ is the sum of the membrane potentials of the neurons in population *m*. *R*_*n*_ is is the sum of spike trains of neurons in population *n*. In other words, its mathematical expectation is the collective firing rate of population *n*. Variable *r*_0_(*t*) is the equivalent firing rate for the external DC input to each neuron, and *N*_*m*_ is the size of the subnetwork *m*. ${L}_{m}(.)$ is a function representing a linear combination of the firing rates of all subnetworks, as well as the external firing rate to each subnetwork *m*. It is easily verified that for a network with fixed subnetwork-specific out-degrees for each neuron, the connectivity matrix *W* for the EEI network is

$${W}_{{\rm{EEI}}}=\tau \varepsilon J(\begin{array}{ccc}w{N}_{E} & {N}_{E} & -pgb{N}_{E}\\ {N}_{E} & w{N}_{E} & -pga{N}_{E}\\ pb{N}_{I} & pa{N}_{I} & -pg{N}_{I}\end{array})$$

where *w* is a factor describing the relative PSP amplitude for couplings within excitatory populations. Inspired by^36^ where they showed that clustered excitatory neurons tend to exhibit stronger EPSP amplitudes, we chose $w=2$ for the simulations. The parameters *a* and *b* are the scaling weight parameters, affecting the strength of neuronal connections. Here, we refer to them as bifurcation parameters. The parameter *g* is the amplitude ratio between IPSPs (inhibitory post-synaptic potentials) and EPSPs (excitatory post-synaptic potentials).

For the EEI network, the structure considered here reflects a scenario where mutual connections between one of the excitatory populations and the inhibitory population is either strengthened or weakened. This could, for example, stand for proportional changes in excitatory and inhibitory synapses in homeostatic plasticity, which maintains the balance between excitation and inhibition, and preserves the asynchronous irregular state in the network dynamics. In particular, *in vivo* studies in CA1 of rats revealed that an increase in mEPSC is accompanied by an enhanced mIPSc^38^. Similarly, excitatory and inhibitory synapses were proportionally modified in rat V1 in response to visual deprivation^39^. This tendency to maintain the balanced condition provides the motivation to scale excitatory and inhibitory synapses by a single parameter (*a* or *b*) in our study.

Other parameters in the model (*g*, *p*, *w*, external input *I*) could also be considered as bifurcation parameters. According to^17^, decreasing *g* can lead the network to a synchronous-regular state, where the balance between excitation and inhibition is disrupted. Likewise, increasing the input *I* can result in the emergence of synchronous-irregular dynamics and fast network oscillations^17^. The coupling and connectivity parameters *w* and *p* influence the functional connectivity between neurons, and increasing these parameters can lead to a different type of asynchronous-irregular state, characterized by strong fluctuations and bursting episodes of neuronal activities^40^.

For the III scenario, we considered the following coupling matrix, according to^37^

$${W}_{{\rm{III}}}=\tau \varepsilon gJ{N}_{I}(\begin{array}{lll}-1 & -a & -b\\ -b & -1 & -a\\ -a & -b & -1\end{array})$$

### Exponential transfer function

To obtain firing rate equations for the activities of subnetworks that are involved in a network, we need to know the dynamic transfer function between the collective membrane potential of each subnetwork and it’s corresponding firing rate. In^10^, it was hypothesized that the response function of a sub-population to the available amount of excitation in the network, in the simple case of a unimodal distribution of synaptic weights, could be well approximated by a sigmoid function (for a more general case, see^18^). A study on transient dynamics of balanced random networks showed that in low firing rate regimes, the distribution of the collective excitatory and inhibitory spike counts follows a log-normal distribution^41^. Assuming in this regime, the neuronal membrane potentials states could be approximated by a normal distribution^17^, this observation implies that an exponential transfer function links the collective membrane potential of each population to its firing rate. Motivated by this idea, here, we assume that if the network is in the low firing rate regime, and the neuronal refractory period can be neglected, the relationship between the collective membrane potential and firing rate of neurons can be approximated by an exponential function

3 $$R=\alpha \,\exp (\beta V).$$

In general, the parameters of this function depend on the size of the neuronal population, as well as on the parameters of the neurons. However, for the sake of simplicity, we assume that they are identical for the three subnetworks in this study.

### Lotka-Volterra equations

Equation (2) together with the exponential relationship between *V*_*s*_ and *R*_*s*_ given by Eq. (3) for a subnetwork *s* yields the following relationships

4 $$\begin{array}{rcl}\frac{d}{dt}{R}_{s} & = & \alpha \beta \frac{d}{dt}{V}_{s}\,\exp (\beta {V}_{s})=\beta {R}_{s}\frac{d}{dt}{V}_{s}\\  & = & {R}_{s}(\,-\frac{1}{\tau }\,\mathrm{log}\,\frac{{R}_{s}}{\alpha }+\frac{\beta }{\tau }{L}_{s}(R)).\end{array}$$

As the coefficients of the linear term are much larger than 1, the logarithmic term in Eq. (4) makes a negligible contribution and will be omitted in our analysis. The resulting equation is a Generalized Lotka-Volterra (GLV) equation. The parameters of the linear function *L*_*a*_(*R*) are inferred from the corresponding connectivity matrix, *W*_EEI_ or *W*_III_. It is important to note that Lotka-Volterra equations represent collective dynamics, regardless of the dynamics of individual nodes. Therefore, the procedure in this paper is to confirm the applicability of such equations, particularly to reduce the dimensionality of the dynamics in networks of leaky integrate-and-fire (LIF) neurons.

#### EEI scenario

**Figure 1:**
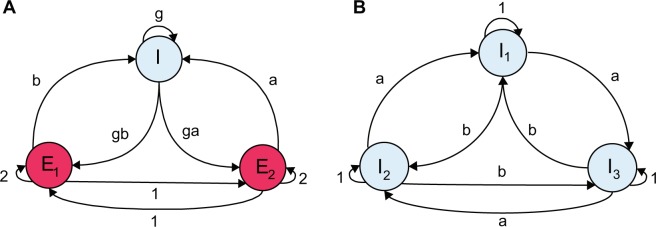
Two scenarios of a network of subnetworks. (**A**) EEI scenario, where one inhibitory and two excitatory populations are interacting. (**B**) III scenario, where all the subnetworks are composed of inhibitory neurons. *a* and *b* are considered as bifurcation parameters. According to Dale’s principle, all connection weights that emanate from inhibitory neurons are negative.

In this case, two excitatory subnetworks of size *N*_*E*_ = 6000 each are reciprocally connected to an inhibitory population of size *N*_*I*_ = 3000 (Fig. 1A). The network is constructed in such a way that for each subnetwork, neurons have identical in-degrees and the number of outgoing connections to each subnetwork are the same (configuration model^42,43^). The rate equations for the involved populations follow

5 $$\begin{array}{rcl}{\dot{x}}_{1}(t) & = & k{x}_{1}(t)\,(2w{x}_{1}(t)+2{x}_{2}(t)-2pgby(t)+2I)\\ {\dot{x}}_{2}(t) & = & k{x}_{2}(t)\,(2{x}_{1}(t)+2w{x}_{2}(t)-2pgay(t)+2I)\\ \dot{y}(t) & = & ky(t)\,(bp{x}_{1}(t)+ap{x}_{2}(t)-pgy(t)+I)\end{array}$$

where *x*_1_ and *x*_2_ are the firing rates of the excitatory populations and *y* stands for the firing rate of the inhibitory population. The variable *R*_*s*_ in Eq. (4) is represented by *x*_1_, *x*_2_ or *y*, depending on the population under study. All network parameters such as network size, time constants and coupling weight *J* that do not show up in Eq. (5) are subsumed by the factor *k* in this equation. As mentioned before, it is assumed that the couplings within each excitatory population are twice stronger than couplings between excitatory neurons in different subnetworks ($w=2$). The factor 2 inside the linear part of the first two equations in (5) reflects the fact that ${N}_{E}=2{N}_{I}$ (the reader is referred to *W*_EEI_ and *W*_III_ in the “Network structure” section). The parameter *I* represents the external input to each population. For simplicity, we consider $I=1$. In comparison with the study in^17^, this reflects the fact that the external input current is close to the rheobase of the network (250 pA), such that it operates in a regime characterized by $\eta \simeq 1$ in^17^. The parameter $k=1$, without loss of generality, represents the time-scale of the system (a new time variable can be defined by rescaling *t*).

To study the time-dependent dynamics and the steady state behavior of the overall network, it is important to analyze the fixed point solutions of Eq. (5) and their stability properties. A three-dimensional GLV, like Eq. (5), typically has 2^3^ = 8 fixed points, corresponding to zero or non-zero solutions of the three dynamical variables *x*_1_, *x*_2_, and *y*. Here, symbolically, we denote the zero and non-zero solutions by 0 and 1, respectively, although non-zero solutions are not necessarily numerically equal to 1. We show the solutions and their corresponding system eigenvalues as a function of the bifurcation parameters *a* and *b*. In the following, we use the parameters $g=6$, $p=3$, and $w=2$.

For the fixed point corresponding to $({x}_{1}^{\ast },{x}_{2}^{\ast },{y}^{\ast })={p}_{1,1,1}$, where *p*_1,1,1_ indicates the fixed point with all nonzero solutions of eq. (5), the parametric solutions are

$$[\begin{array}{l}{x}_{1}^{\ast }\\ {x}_{2}^{\ast }\\ {y}^{\ast }\end{array}]=\frac{-I}{3(\,-\,2{a}^{2}+2ab-2{b}^{2}+1)}[\begin{array}{c}a-2b+3ab-3{a}^{2}+1\\ b-2a+3ab-3{b}^{2}+1\\ \frac{a+b-1}{6}\end{array}].$$

In order to study the local stability of this fixed point, the eigenvalues of Jacobian matrix evaluated at the fixed point need to be considered. The Jacobian matrix for *p*_1,1,1_ is

$$Ja{c}_{111}=[\begin{array}{ccc}8{x}_{1}^{\ast }+2{x}_{2}^{\ast }-36b{y}^{\ast }+2I & 2{x}_{1}^{\ast } & -36b{x}_{1}^{\ast }\\ 2{x}_{2}^{\ast } & 2{x}_{1}^{\ast }+8{x}_{2}^{\ast }-36a{y}^{\ast }+2I & -36a{x}_{2}^{\ast }\\ 3b{y}^{\ast } & 3a{y}^{\ast } & 3b{x}_{1}^{\ast }+3a{x}_{2}^{\ast }-36{y}^{\ast }+I\end{array}].$$

**Figure 2:**
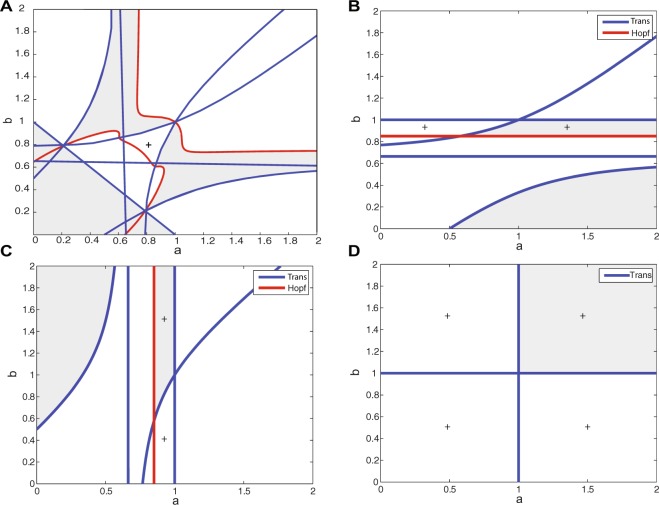
Local stability analysis of fixed points in the EEI scenario. Stable regions for (**A**) *p*_1,1,1_, (**B**) *p*_1,0,1_, (**C**) *p*_0,1,1_ and (**D**) *p*_0,0,1_ are depicted in gray. The corresponding fixed point in the white regions has at least one unstable manifold (at least one eigenvalue has a positive real part). In each case, Hopf and transcritical bifurcations are depicted in red and blue, respectively. Regions with a (+) show that the fixed point is in the positive octant of the state space.

Numerical continuation method of integration^44^ for these equations show that for *p*_1,1,1_, there is a supercritical Hopf bifurcation that passes through *p*_0,0,1_. This bifurcation line is illustrated in red in Fig. 2A.

For the solution $({x}_{1}^{\ast },{x}_{2}^{\ast },{y}^{\ast })={p}_{0,1,1}$, it is easy to get

$$[\begin{array}{l}{x}_{1}^{\ast }\\ {x}_{2}^{\ast }\\ {y}^{\ast }\end{array}]=[\begin{array}{c}0\\ \frac{I(1-a)}{3{a}^{2}-2}\\ \frac{I(3a-2)}{18(3{a}^{2}-2)}\end{array}].$$

The eigenvalues of the Jacobian at this fixed point are

6 $$\begin{array}{rcl}{\lambda }_{1} & = & \frac{2I(a-2b+3ab-3{a}^{2}+1)}{2-3{a}^{2}}\\ {\lambda }_{2,3} & = & \frac{I(6-7a\pm \sqrt{72{a}^{4}-120{a}^{3}+49{a}^{2}-4a+4})}{6{a}^{2}-4}\end{array}$$

To obtain the transcritical bifurcation lines^45^ in the parameter space, one needs to solve for ${\lambda }_{1}=0$ and ${\lambda }_{2,3}=0$. The former results in $b=\frac{3{a}^{2}-a-1}{3a-2}$, which is a curve in the *a*–*b* plane. The latter will result in $a=1$ and *a* = 2/3 for the transcritical bifurcation lines, and $a=0.8571$ for a degenerate Hopf bifurcation. Due to the symmetry between *x*_1_ and *x*_2_ in the equations, it is easy to get the solutions for the fixed point $({x}_{1}^{\ast },{x}_{2}^{\ast },{y}^{\ast })={p}_{1,0,1}$. In this case, $a=\frac{3{b}^{2}-b-1}{3b-2}$, as well as $b=1$, *b* = 2/3, and $b=0.816$ determine a transcritical bifurcation line. The line $b=0.8571$ represents a degenerate Hopf bifurcation for this fixed point.

For $({x}_{1}^{\ast },{x}_{2}^{\ast },{y}^{\ast })={p}_{0,0,1}$, it turns out that the values of the parameters do not play any role in determining the fixed point which is (0, 0, *I*/18). The corresponding eigenvalues of the Jacobian are

7 $$\begin{array}{rcl}{\lambda }_{1} & = & -I\\ {\lambda }_{2} & = & -2I(a-1)\\ {\lambda }_{3} & = & -2I(b-1)\end{array}.$$

This indicates that for *a*, *b* > 1, the fixed point is locally stable.

For the origin $({x}_{1}^{\ast },{x}_{2}^{\ast },{y}^{\ast })={p}_{0,0,0}=(0,0,0)$, the eigenvalues are ${\lambda }_{1}=I$, and ${\lambda }_{2,3}=2I$, which are always positive in spiking neural networks with a positive external input. Furthermore, for $({x}_{1}^{\ast },{x}_{2}^{\ast },{y}^{\ast })={p}_{1,1,0}$, the parametric solutions are $(\,-\,I/3,-\,I/3,0)$. For $({x}_{1}^{\ast },{x}_{2}^{\ast },{y}^{\ast })={p}_{1,0,0}$ and ${p}_{0,1,0}$, the parametric solutions are $(\,-\,I/2,0,0)$ and $(0,-\,I/2,0)$, respectively. The last three cases are impossible solutions for the firing rates of spiking neuronal networks. Therefore, we will not consider their stability and their influence on the trajectory of the network.

#### III scenario

This system represents a neuronal implementation of the May-Leonard equation^37^, which is well-known for generating oscillatory population dynamics for some parameter ranges. As depicted in Fig. 1B, the scaling factor for the coupling weights is *a* for clockwise connections, and *b* for counterclockwise couplings. In this case, each inhibitory subnetwork comprises of 4000 neurons. The corresponding equations for the GLV dynamics are

8 $$\begin{array}{rcl}{\dot{x}}_{1}(t) & = & k{x}_{1}(t)\,(\,-\,{x}_{1}(t)-a{x}_{2}(t)-b{x}_{3}(t)+I)\\ {\dot{x}}_{2}(t) & = & k{x}_{2}(t)\,(\,-\,b{x}_{1}(t)-{x}_{2}(t)-a{x}_{3}(t)+I)\\ {\dot{x}}_{3}(t) & = & k{x}_{3}(t)\,(\,-\,a{x}_{1}(t)-b{x}_{2}(t)-{x}_{3}(t)+I)\end{array}$$

where *x*_1_, *x*_2_ and *x*_3_ represent the firing rates of the three inhibitory subnetworks, respectively. Similar to the EEI case, we assume $I=1$. A full bifurcation analysis for this system of equations is given in^37^. For $a+b < 2$, each population has a stable equilibrium value at $\frac{1}{1+a+b}$. For $a+b > 2$, and when both *a*, *b* > 1, all three fixed points denoted by *p*_1,0,0_, *p*_0,1,0_, *and p*_0,0,1_ are stable. Depending on the initial condition, one population wins the competition, and the system exhibits a winner-take-all (WTA) dynamics. However, if either $a < 1$ or $b < 1$, no stable equilibrium exists, and an oscillatory solution emerges. For $a+b > 2$, asymptotically, the solution lies on the plane determined by ${x}_{1}+{x}_{2}+{x}_{3}=1$, and the condition ${x}_{1}\,{x}_{2}\,{x}_{3}={\exp }^{-(a+b-2)t}$ is satisfied. It was shown in^37^ that under these conditions, the time that the trajectory spends near each of the fixed points is proportional to the total time elapsed until the network reached that state. This entails an oscillatory solution where the period increases linearly with time.

## Results

In this section, we compare our analytical treatment of the GLV equations with spiking network simulations for both examples considered in this paper, i.e. EEI and III networks. All numerical simulations were conducted in NEST^46^ for a duration of 4 seconds.

### EEI scenario

For a generic three-dimensional Lotka-Volterra equation, 2^3^ = 8 different fixed points are possible. However, in the Lotka-Volterra system with *W*_EEI_ connectivity matrix, three of the fixed points have negative components regardless of the choice of parameters *a* and *b*, excluding them as a biological firing rate. Consequently, with initial conditions chosen in the positive octant, only the fixed points in this octant need to be considered. The reason is that in a GLV system, trajectories remain non-negative if the initial conditions have this property. Moreover, in Eq. (5) the origin always represents an unstable fixed point. Therefore, we only analyze the system dynamics for four different fixed points $({x}_{1},{x}_{2},y)$, denoted by *p*_1,1,1_, *p*_1,0,1_, *p*_0,1,1_, *p*_0,0,1_. Here, 0 means no activity and 1, symbolically, means that the corresponding population is active and has a non-zero firing rate. Note that Eqs (5) and (8) did not aim at capturing the exact solutions of the network dynamics in terms of steady state values and time constants, but rather represent the qualitative behavior of the dynamics as the bifurcation parameters are changed.

#### Stability analysis

Figure 2 depicts the locally stable regions in the parameter space, where all eigenvalues have a negative real part (gray shaded area), for each of the above-mentioned fixed points. Moreover, Hopf and transcritical bifurcation lines for each fixed point are represented in red and blue, respectively (The numerical bifurcation analysis was performed in MATCONT v6.1^44^). A + sign in a region indicates that the corresponding fixed point is in the first octant.

The straight lines $a=0.8571$ and $b=0.8571$ are generalized Hopf bifurcation for *p*_0,1,1_ and *p*_1,0,1_, respectively. This bifurcation is not generic, however, since the second Lyapunov exponent is zero. Numerical analysis shows that for these parameter values, in the first octant and on the ${x}_{1}=0$ plane or ${x}_{2}=0$ plane, there is one saddle limit cycle, as there are two Floquet multipliers (eigenvalues of the discrete map for the cycle) equal to −1 and +1. As the value of the parameter increases, the trajectory will converge to a stable fixed point on the plane; meaning, depending on the initial condition, the trajectory converges either to *p*_1,0,1_ or to *p*_0,1,1_.

**Figure 3:**
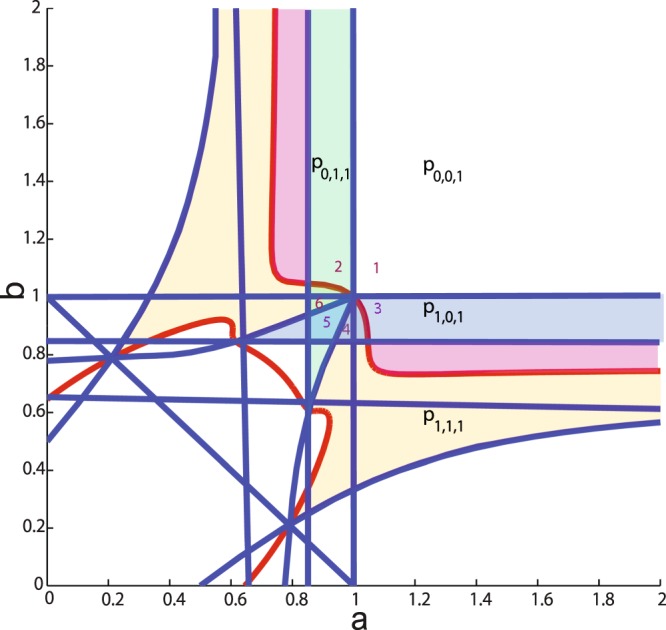
Local stability of fixed points in the parameter space. For the region of interest, the stability of different fixed points is highlighted with different colors. See text for more details.

**Figure 4:**
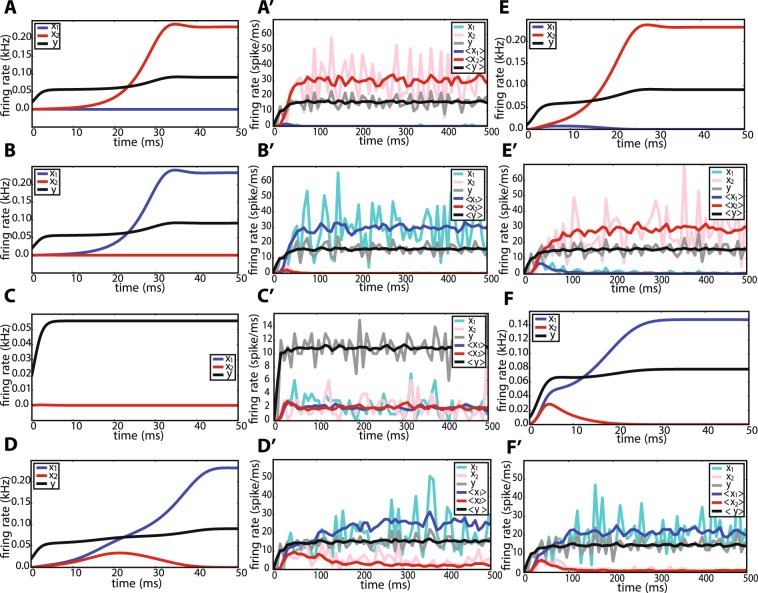
Numerical integration of Eq. (5) and subnetwork firing rates for different parameters corresponding to the 6 regions. (**A**) For $a=0.9$, $b=1.3$ (region 2) and initial conditions $(0.0001,0.0001,0.02)$, the trajectory passes by the vicinity of *p*_0,0,1_, and eventually converges to *p*_0,1,1_. (**B**) For $a=1.2$, $b=0.9$, and $(0.0001,0.0001,0.02)$ as the initial condition, the trajectory follows a heteroclinic connection between *p*_0,0,1_ and *p*_1,0,1_. This parameter combination corresponds to region 3. (**C**) For $a=b=1.2$, and any initial condition in the first octant, the trajectory converges to *p*_0,0,1_. (**D**) For $a=b=0.9$ (region 5) and initial conditions equal to $(0.0004,0.0003,0.02)$, the trajectory passes by the points *p*_0,0,1_ and *p*_1,1,1_, and eventually converges to *p*_1,0,1_. In this case, a longer heteroclinic connection between saddle points exists. For initial conditions with ${x}_{2} > {x}_{1}$, the trajectory will converge to *p*_0,1,1_. (**E**) For $a=0.90$, $b=0.97$, and initial condition $(0.0002,0.0002,0.01)$, the trajectory represents a heteroclinic connection between *p*_0,0,1_ and *p*_1,0,1_, and will finally converge to *p*_0,1,1_. The amount of time that the trajectory spends close to the saddle points, and also how close it gets to each saddle point, depends on the initial conditions. This parameter combination corresponds to region 6. (**F**) For $a=0.98$ and $b=0.92$ which corresponds to region 4, and the initial condition $(0.001,0.001,0.01)$, the trajectory follows a heteroclinic connection between *p*_0,0,0_, *p*_0,0,1_ and *p*_0,1,1_, and eventually converges to *p*_1,0,1_. (**A′**–**F′**) Firing rates of different subnetworks for each parameter combination in (**A**–**F**), respectively. Mean firing rates over 20 trials for each case and each subnetwork is plotted in darker colors.

**Figure 5:**
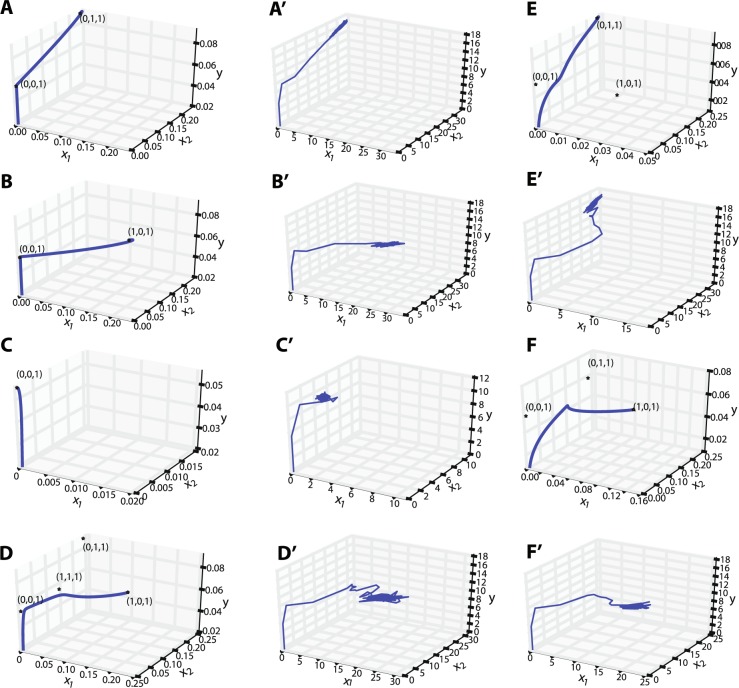
Three-dimensional trajectories in the population variable state space for different parameters corresponding to the 6 regions. (**A**–**F**) Trajectories that result from numerical integration of Eq. (5) for each parameter combination in Fig. 4, respectively. (**A′**–**F′**) Firing rate trajectories in a three-dimensional state space corresponding to spiking network simulations in Fig. 4A′–F′, respectively.

We now focus on the parameter region *a*, $b > 0.8571$, because only under this condition neurons operate in the fluctuation-driven regime, indicated by network simulations. Different bifurcation lines of the fixed points divide the parameter space into six different regions (Fig. 3). In region 1, *p*_0,0,1_ is a globally stable fixed point, and therefore, all trajectories will end in this point. Figures 4C and 5C illustrate a trajectory in the time domain and the state space in this parameter space.

In regions 2 and 3 in Fig. 3, apart from the origin *p*_0,0,0_ and *p*_0,0,1_, there exist also *p*_0,1,1_ and *p*_1,0,1_, respectively. In these regions, there is a heteroclinic connection between *p*_0,0,0_ to *p*_0,0,1_. The only unstable manifold of *p*_0,0,1_ is directed to the stable manifold of *p*_0,1,1_ in region 2 (Fig. 4A), and to the stable manifold of *p*_1,0,1_ in region 3 (Fig. 4B). Thus, depending on the initial conditions, the trajectory passes by a few number of saddle points and eventually settles in a globally stable fixed point.

**Figure 6:**
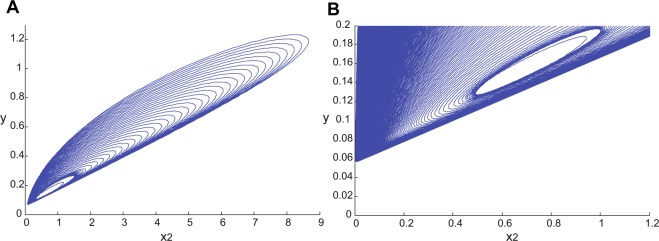
Homoclinic connection for *p*_0,0,1_ on the *x*_1_ = 0 plane. (**A**) Limit cycles that bifurcate from *p*_0,1,1_ at $a=0.8571$ become tangent to *p*_0,0,1_ when the period of the cycle tends to infinity. (**B**) Zoom into the area of interest, close to the point $(0,0,0.0556)$ which is the fixed point of the GLV corresponding to *p*_0,0,1_.

In regions 4, 5 and 6, the fixed point *p*_0,0,1_ has a two-dimensional unstable and a one-dimensional stable manifold with real eigenvalues. The directions of the eigenvectors corresponding to the unstable eigenvalues point towards the other fixed points *p*_0,1,1_ and *p*_1,0,1_ and build a heteroclinic connection (Figure 4D–F). The *y* axis is the stable manifold. The saddle value, i.e., the sum of the real parts of the closest eigenvalues to the imaginary axis on the right and left side of the axis, for this fixed point is always negative, in the range of interest for *a* and *b*. As pointed out earlier, *a* = 0.8571 and *b* = 0.8571 are generalized Hopf bifurcation curves for *p*_0,1,1_ and *p*_1,0,1_, respectively. From our numerical analysis it turns out that the limit cycles that bifurcate from these fixed points touch the *p*_0,0,1_ fixed point when the period of the cycle tends to infinity (Fig. 6). Therefore, *a* = 0.8571 and *b* = 0.8571 are homoclinic bifurcation curves for *p*_0,0,1_ as well. In this parameter range, the saddle value is negative. According to Shilnikov’s theorem for two unstable–one stable manifold in a three-dimensional system (see^47^), the limit cycles that bifurcate from this curve should, under generic conditions, generate a saddle limit cycle in the vicinity of ${x}_{1}=0$ and ${x}_{2}=0$ planes when *a* and *b* increase. However, the Hopf bifurcation is degenerate and only on the bifurcation lines $a=0.8571$ and $b=0.8571$, a homoclinic connection exists. Therefore, as the values of the parameters are changed away from the bifurcation lines, any trajectory will end up in the corresponding fixed point.

In region 5, depending on the initial conditions, the trajectory will either converge to *p*_0,1,1_ or *p*_1,0,1_. For $a=0.9$ and $b=0.9$, for example, both fixed points have two complex conjugate eigenvalues with negative real parts, and one negative real eigenvalue. Moreover, in this region, *p*_1,1,1_ is in the first octant and has one positive real eigenvalue for which the corresponding unstable manifold points towards the fixed points *p*_1,0,1_ or *p*_0,1,1_ (Fig. 4D). Depending on possible fluctuations in network activity, the trajectory would eventually converge to either of these two fixed points.

In region 6, for $a=0.9$ and $b=0.97$ for example, *p*_0,1,1_ has three eigenvalues with negative real parts, two of which are complex conjugate. This indicates the local stability of this point. The point *p*_1,0,1_ has three real eigenvalues, two negative and one positive eigenvalue. In fact, the latter has changed sign due to a bifurcation, as the value of *b* was increased. In this region, *p*_0,0,1_ still has two positive real eigenvalues and one negative real eigenvalue, and *p*_1,1,1_ is not in the first octant. The fixed point *p*_1,0,1_ has a positive eigenvalue which creates an unstable manifold in the direction of the only attractor in the first octant, namely *p*_0,1,1_. Putting all this information together, one concludes that there is a heteroclinic chain for the trajectory, starting in *p*_0,0,0_ and then moving to *p*_0,0,1_, and after a slight detour toward *p*_1,0,1_ finally heads toward *p*_0,1,1_ (Figs 4E and 5E). For initial conditions close to *p*_1,0,1_, the trajectory will be lead to *p*_0,1,1_ via the unstable manifold of *p*_1,0,1_. Due to the existing symmetry between *a* and *b* in the parameter space, the same behavior applies to region 4, but with the relevant fixed points interchanged (Figs 4F and 5F).

#### Network simulation

**Figure 7:**
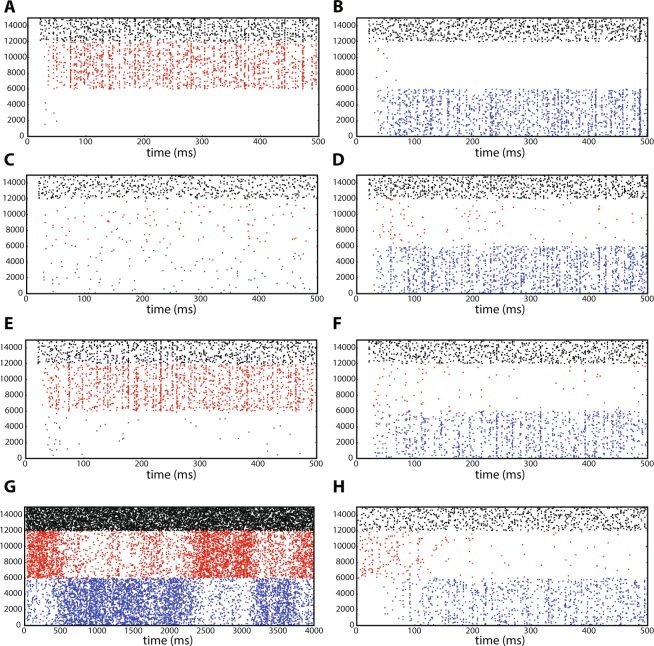
Raster plots of the network activity for the EEI scenario. (**A**) For $a=0.9$ and $b=1.3$, the second excitatory population dominates the activity of the first excitatory population. (**B**) For $a=1.2$ and $b=0.9$, the first excitatory population is more active than the second excitatory population. (**C**) For $a=1.2$ and $b=1.2$, the only strongly active population is the inhibitory subnetwork. (**D**) For $a=0.9$ and $b=0.9$, the two excitatory populations compete with each other. It depends on the initial fluctuations of the network dynamics which population wins the competition. (**E**) At $a=0.90$ and $b=0.97$, the network activity is such that the inhibitory population is active together with the second excitatory population. (**F**) For $a=0.98$ and $b=0.92$, after a brief period of the activity of the inhibitory subnetwork, the two excitatory populations also become active. However, the second excitatory population is much less active compared to the first excitatory population. (**G**) For $a=b=0.92$, a switching dynamics between the two excitatory populations is observed. (**H**) For $a=0.98$ and $b=0.92$, starting with an initial condition that favors the second excitatory population, after a short transient, the network activity converges to a state in which the first excitatory population as well as the inhibitory population exhibit higher activity.

We performed numerical simulations of networks of leaky integrate-and-fire neurons for samples of parameter combinations (*a*, *b*) from the six regions defined in the previous section. Raster plots of the two populations of excitatory neurons are shown in blue and red corresponding to *x*_1_ and *x*_2_, respectively, in Fig. 7. The activity of neurons from the inhibitory population are shown in black. The index of the neurons on the vertical axis are between 1 and 15000, the first 6000 neurons belong to the first excitatory population. Neurons with an index between 6001 and 12000 belong to the second excitatory population, and neurons with indices between 12001 and 15000 correspond to the inhibitory population. In all of the sub-figures, apart from panel G, after a short transient, the network activity remains in a steady state, which is stable apart from quasi-stochastic fluctuations.

In Fig. 7 the initial conditions for the neuronal membrane potentials were randomly chosen between the threshold value at $0\,{\rm{mV}}$ and $15\,{\rm{mV}}$, for the excitatory neurons. The initial conditions for neurons in the inhibitory subnetwork were chosen between 0 and $17\,{\rm{mV}}$ (the threshold level is at $20\,{\rm{mV}}$ for all neurons). In a reduced-dimensional model, this would correspond to an initial condition close to *p*_0,0,0_. For details on the initial conditions and the model behavior, see Fig. 4. In Fig. 7A, for $a=0.9$ and $b=1.3$, in the first $25\,{\rm{ms}}$ of the network simulation, there is a small episode within which only the inhibitory population is active. Then, there is a very brief activity of the first excitatory population, together with a more prominent activity of the second excitatory population. Afterwards, the steady state activity is such that the inhibitory population together with the second excitatory population remain active, and the first excitatory population becomes silent. The firing rates of individual populations calculated in time bins of 8 ms for a single trial, as well as the subnetwork firing rates averaged over 20 trials are illustrated in Fig. 4A′. This nicely matches the behavior of the trajectory in region 2, displayed in Fig. 4A. Moreover, the three-dimensional state trajectory for the subnetwork firing rates represents the expected behavior very well (Fig. 5A and A′), as the trajectory initiated from *p*_0,0,0_ initially moves toward increasing the inhibitory firing rate, and after passing by the associated saddle point, it heads toward the stable fixed point represented by a high value of *x*_2_ and a small value of *x*_1_. In Fig. 7B′, for $a=1.2$ and $b=0.9$, the activity in the transient phase corresponds to an initial condition close to *p*_0,0,1_ (as explained before). After a short time, the inhibitory and first excitatory subnetwork are highly active, and the second excitatory subnetwork remains silent. This was also expected from the GLV analysis (Fig. 4B). In Fig. 7D, *a* and *b* are both set to 0.9. The GLV analysis indicates that the saddle fixed point *p*_1,1,1_ is localized in the first octant. The network activity is such that after a high activity of the inhibitory population and the extended silence period of the two excitatory populations for almost 10 ms, the network activity switches to a state wherein all three populations are active, corresponding to the saddle point *p*_1,1,1_. After about 150 ms, the network activity settles in a steady state, where the inhibitory subnetwork together with the first excitatory subnetwork are active, and the second excitatory subnetwork has a very low activity (Fig. 4D′ shows the average firing rates over 10 trials in dark colors). This corresponds to the stable fixed point *p*_1,0,1_ in the GLV equations (note that Fig. 5D′ exhibits a similar trajectory as the one shown in Fig. 5D, in which two saddle points may be present). In repeated network simulations, we observed that the steady state could also correspond to *p*_0,1,1_, with equal likelihood. Also, for parameters *a* and *b* close to 1, the network can exhibit bistable dynamics (see^26^ for more details). For instance, for $a=b=0.92$, the two excitatory subnetworks enter a switching dynamics in their activity, which is illustrated in Fig. 7G. A similar behavior was described in^26^ where the existence of two attractors, separated by a saddle point, lead to a similar switching behavior realized by fluctuating firing rates (see also^48^).

For $a=b=1.2$, it was observed that the inhibitory neuronal population exhibits a considerably higher firing rate as compared to the excitatory subnetworks (Fig. 7C). GLV integration results also illustrate a unique stable fixed point in the first octant, that is *p*_0,0,1_ (Figs 4C and 5C). A three-dimensional state space plot indicates that the trajectories of average firing rates converge to a stable fixed point which is characterized by a high inhibitory and low excitatory firing rates (Fig. 5C′). If the parameters *a* and *b* are increased further, the excitatory firing rates decrease more, and the three-dimensional trajectory looks even more similar to Fig. 5C.

As an example corresponding to region 6 in Fig. 3, we chose $a=0.9$ and $b=0.97$ (see Fig. 7E for the raster plots and Fig. 4E′ for the firing rates). The initial conditions were chosen such that in the low-dimensional system, the network is close to *p*_0,0,0_. It was observed that, after passing by two saddle points, the activity of the network reaches a steady state, where the inhibitory population and the second excitatory population are active (corresponding to *p*_0,1,1_, which is the stable fixed point in region 6). This result is confirmed by GLV equations (Fig. 5E and E′). For an example that corresponds to region 4, we chose $a=0.98$ and $b=0.92$. For this case, we considered two different initial conditions for the network simulations. First, the membrane potentials of all neurons in the two excitatory populations were initialized randomly (uniform distribution) between 0 and 15 mV, and the inhibitory neurons were chosen to have initial conditions between 0 and 17 mV. Under this condition, the raster plot shows that initially only the inhibitory population is activated for a brief period, followed by activity of both excitatory populations. Then, the activity converges to a state where only the first excitatory population as well as the inhibitory population are active (Fig. 4F′, 5F′ and 7F). The transient dynamics in Fig. 5F′ indicate the possible existence of two saddle points (similar to Fig. 5F) than bend the trajectory before it reaches the stable fixed point. Second, to illustrate the behavior of the network with a different initialization far from the stable fixed point, the initial conditions for the membrane potentials of the first excitatory population (which will take a high value at the steady state according to the mathematical analysis), were chosen randomly in the range between 0 and 15 mV. The membrane potentials of the inhibitory and the second excitatory populations, respectively, were initially set to a random value between 0 and 20 mV. As indicated in Fig. 7H, and as predicted by the GLV equations in Fig. 5F′, the activity transient corresponding to *p*_0,1,1_ will eventually settle in *p*_1,0,1_, meaning that the steady state of the network would have a highly active inhibitory and first excitatory subnetwork.

**Figure 8:**
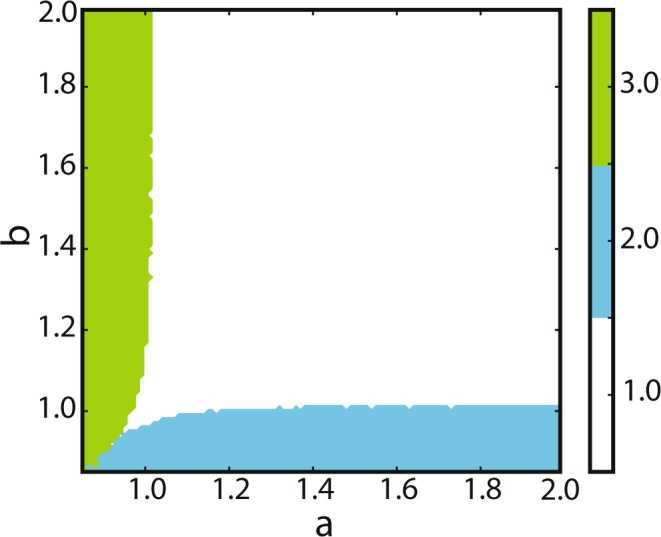
Bifurcation diagram of the collective activity of the network in the EEI scenario, extracted from numerical simulations. This plot is a result of a three-state classification based on projections on the vectors $(0,0,1)$, $\frac{1}{\sqrt{2}}(0,1,1)$, and $\frac{1}{\sqrt{2}}(1,0,1)$, corresponding to regions 1–3 in Fig. 3. The color code stands for different regions, labeled as 1, 2, and 3 in Fig. 3. The bistable region is reflected by sporadic pixels of different colors around the line $a=b$, when both parameters are below 1.

For *a* and *b* between 0.85 and 2.0, we performed network simulations for a duration of 4 seconds, with a simulation time step of 0.1 ms. After discarding the network transients to the steady state (the first 100 ms), we calculated the average neural firing rate for each subnetwork in the stationary state. As the fixed points in the first octant correspond to one of the points *p*_0,0,1_, *p*_0,1,1_ or *p*_1,0,1_, we chose three vectors of length 1 to represent these network states, namely (0, 0, 1), $(0,\frac{1}{\sqrt{2}},\frac{1}{\sqrt{2}})$, and $(\frac{1}{\sqrt{2}},0,\frac{1}{\sqrt{2}})$, respectively. To classify the emerging network states as a function of the bifurcation parameters, we calculated the projections of the vector representing the network activity in the steady state on these representative vectors. The network state was then assigned to the class with the largest projection. We plotted these winning states as a function of bifurcation parameters *a* and *b* in Fig. 8. This plot provides sufficient information about the activities of the sub-populations, and therefore a classification of the network dynamics. For values of *a* and *b* close to 1, the average firing rate of inhibitory neurons is large compared to the firing rates of excitatory neurons. The bifurcation diagram of the network, obtained from network simulations, can be inferred from this classification, as each network state corresponds to a different fixed point. As presented in Fig. 8, the separation between classes, which can be interpreted as bifurcation lines (because they correspond to a different collective behavior), have a similar shape as those of Fig. 3. The reason is that in the specified parameter range, mostly transcritical bifurcations occur. This results in a change of stability of the fixed points, reflected by a change of the firing rates of the neuronal populations. Compared to Fig. 3, it is clear that in the fluctuation driven regime, GLV systems can represent spiking network dynamics with high fidelity.

In the bifurcation plot that was inferred from simulations, the region with $a > 1$, and $b > 1$ was associated with the vector $(0,0,1)$ representing *p*_0,0,1_. For *a*, *b* slightly less than 1, this fixed point was still dominant, however this region was very small, and its existence is probably due to the finite size of the network (see section III for a similar discussion). As predicted by theory, the bifurcation lines that separate *p*_0,1,1_ from *p*_1,0,1_ in Fig. 8 are very similar to those shown in Fig. 3. Note that regions 2 and 6 have the same attractor, but they differ by their transitions towards the fixed point. The same holds for regions 3 and 4. If $a < 1$ and $b < 1$ are very close to each other, the collective behavior of the network depends on the initial conditions. This is exactly the region where a linearization about *p*_0,0,1_ has two positive eigenvalues in the GLV model, which allows the trajectory to converge into any of the stable fixed points (corresponding to region 5 in Fig. 3), or into the unique stable fixed point (corresponding to regions 4 or 6). However, the bistable region 5 in Fig. 3 is larger than what is shown in Fig. 8. As a matter of fact, the similarity between spiking network simulations and the theoretical predictions increases as the network size approaches infinity, because the theory is valid under this condition.

### III scenario

**Figure 9:**
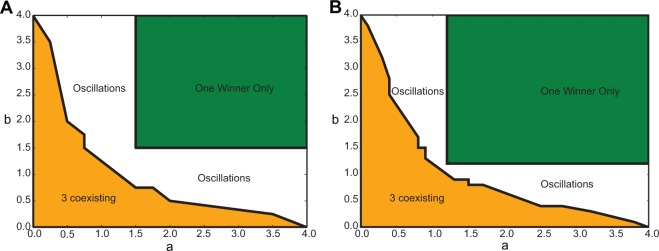
Bifurcation diagram for the network dynamics in the III scenario. Bifurcation diagram obtained from numerical simulations of a spiking network composed of (**A**) 12000 inhibitory LIF neurons, divided into three subnetworks of equal size, and (**B**) 24000 inhibitory LIF neurons, divided into three identical subnetworks, illustrates three different types of collective dynamics. Depending on the parameter combinations, either all subnetworks are active simultaneously (orange region), or only one population is active and others have a zero firing rate (green region), or the firing rates of the populations follow an oscillatory pattern (white region).

To demonstrate the power of GLV equations in representing spiking network dynamics, we also considered a purely inhibitory network that represents a May-Leonard competitive system^37^. We chose a synaptic coupling equal to $J=-\,0.012\,{\rm{mV}}$, but the network state is similar for larger values of *J*. The connection probability was 0.1 throughout, and we considered networks with identical in-degrees and identical out-degrees for all neurons in order to exclude any structural bias for the network dynamics. The bifurcation diagram obtained by May and Leonard^37^ through theoretical analysis has the following properties: (1) For $a+b < 2$ the three inhibitory populations are active with equal rates. (2) For $a > 1$ and $b > 1$, only one population is active. (3) For any other parameter combination, the network shows oscillations in the firing rates of the three populations. The period of oscillations increases as a function of time; however, for $a+b=2$, a stable limit cycle solution exists. For details of the mathematical analysis, see^37^. Figure 9 is obtained from spiking network simulations of a network with 3 inhibitory subnetworks, each of them composed of 4000 neurons (panel A), and a network with twice as many neurons (8000 neurons in each subnetwork) in panel B. There is a very good match between the two diagrams (Fig. 9), and a similar diagram obtained by theoretical analysis in^37^. For small values of *a* and *b*, in the orange region of the diagrams shown, all three neuronal populations are active, with non-zero firing rates. In the example of the smaller network (with 4000 neurons in each population), for values of *a* and *b* larger than 1.5, only one population has a non-zero firing rate. In the diagrams obtained from network simulations, there is a gap between the green and the orange region that should in theory disappear at *a*, *b* = 1 (similar to^37^). Our simulations indicate that with increasing network size, these small discrepancies vanish. Comparing Fig. 9A,B supports this claim by demonstrating that the origin of the winner-take-all dynamics for the smaller network is at $(a,b)=(1.5,1.5)$, while this origin for the bigger network is located at $(a,b)=(1.2,1.2)$. This behavior is expected, as the GLV equations become a better approximation for the dynamics of a network with a very large (ideally infinite) number of neurons, because in this limit, among other things, the birth and death rates for the population dynamics can be more accurately described. Therefore, expressing the population dynamics by ordinary differential equations that have these rates as parameters will be more accurate in the large size limit. For the parameter combinations in the white region of the figure, the dynamical behavior is different from any of the aforementioned cases, and an oscillatory dynamics was observed.

**Figure 10:**
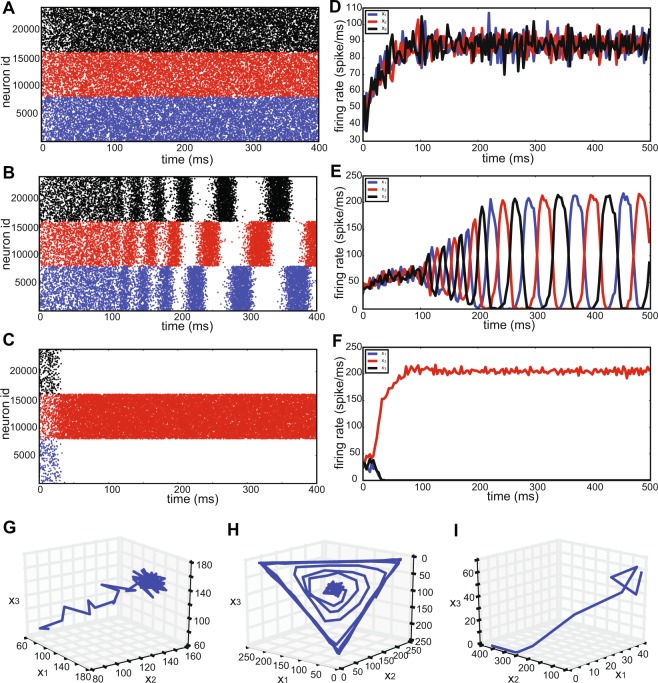
Sample simulations of a large spiking network in the III scenario. Three different samples of spiking network simulations are shown, corresponding to the three different dynamical behaviors of the III network with 24000 neurons. (**A**) $a=0.75$, $b=0.75$, where all the populations are active. (**B**) $a=1.4$, $b=1.0$, where the subnetworks oscillate with a phase difference of 2*π*/3. (**C**) $a=2.0$, $b=2.0$, where only one inhibitory population is active, persistently dominating the other two populations. (**D**–**F**) Population firing rates corresponding to the neural spiking dynamics in (**A**–**C**), respectively, obtained by counting the spikes generated by each subnetwork in time bins of width 3 ms. (**G**) State space embedding of the firing rates of the three subnetworks for $a=0.75$, $b=0.75$. The activity fluctuates around a fixed point that represents a stable equilibrium. (**H**) State space illustration of the firing rates of the three subnetworks for $a=1.4$, $b=1.0$. In this case, oscillations of increasing period emerge, and the state space representation is a closed trajectory, after a long enough time has elapsed. (**I**) State space illustration of the firing rates of the three subnetworks for $a=2.0$, $b=2.0$, where the trajectory converges to a single attractive fixed point and remains there.

There are three regions in the bifurcation diagram with different dynamics that are confirmed by simulation results in Fig. 10, which illustrates one example for each possible network dynamics. Note that in panel C of this figure, depending on the initial conditions, the system has only one active inhibitory population, and all other populations remain silent. Corresponding firing rates for each network example are demonstrated in Fig. 10D–F. In these figures, the spike counts of each subnetwork in time bins of width 3 ms are plotted. Moreover, the firing rate trajectories in the 3-dimensional state space representing the three subnetworks, for each example shown in panels A-C, are depicted in Figure 10G–I. This plot illustrates the full repertoire of different network dynamics of this system.

In simulations for the parameter region where oscillations occur, we made the interesting observation that the period of the high activity of the three inhibitory populations increases as a function of time, following the initial transients (Fig. 10B). However, the period does not appear to grow without bounds. We hypothesize that this phenomenon is due to finite-size fluctuations in the spiking network dynamics, which is not captured by the GLV. In other words, in simulations, the population firing rate exhibits excursions that randomly deviate from the deterministic GLV solutions. This keeps the simulated trajectories to get very close to the heteroclinic cycle, avoiding the critical slowing down that would otherwise result. Therefore, the period of the oscillations cannot increase beyond a bound that is related to the amplitude of fluctuations of the network activity. This constrains the joint firing rates to a certain region in the state space, and does not allow any growth beyond this limit.

## Discussion

We studied dynamical interactions between subnetworks of different types of neurons, within a large network. For two example networks, we focused on the role of the strength of couplings between subnetworks for global network dynamics. Such a study can shed new light, for example, on the interaction dynamics between different brain nuclei in the basal ganglia, or between “columns” or cell assemblies in a certain region of neocortex. Applications of such mathematical modeling would, among other things, help to predict aberrant network dynamics that underlies certain brain disorders, as described in Parkinson’s Disease or certain types of epilepsy.

In our study, the dynamics of networks comprising interacting subnetworks of excitatory and inhibitory neurons were compared with the dynamics of ecosystems comprised of prey and predator species, corresponding to the excitatory and inhibitory subnetworks, respectively. Specifically, we considered Generalized Lotka-Volterra (GLV) systems and numerical simulations of spiking networks composed of three subnetworks, with leaky integrate-and-fire (LIF) neurons as dynamical nodes. In both cases, coupling strength was conceived as a bifurcation parameter. Bifurcation diagrams extracted from the GLV systems and from the numerical simulations were strikingly similar in the two examples we studied. This indicates that GLV represents a meaningful model of competing populations of spiking neurons, and there is a qualitative equivalence between the mathematical equations and the behavior of the simulated network. This qualitative equivalence is a consequence of the topological equivalence of the two systems that results in similar bifurcation diagrams. In the model considered here, it was possible to predict convergence towards the correct fixed points (as validated by the simulation results), as well as oscillatory dynamics in the purely inhibitory network, for the correct parameter regime. The EEI network studied here is an extension of a similar study^26^ in which GLV was envisaged to explain the emergence of switching dynamics (and also different network states) for a special choice of $(a,b)=(1,1)$. Our study shows that this parameter configuration results in a degenerate dynamics due to emergence of two zero eigenvalues for the relevant fixed point of the system (*p*_0,0,1_). Moreover, for this choice of parameters, the other two fixed points, namely *p*_0,1,1_ and *p*_1,0,1_ merge into *p*_0,0,1_. In other words, the EEI network in^26^ is a special case of the EEI scenario that we studied here, where the effect of couplings within each excitatory subnetwork was studied. In both studies, GLV equations have successfully described the emergent global network dynamics under parameter changes for different networks of interacting subnetworks.

Whether GLV could represent a model for any network of arbitrary number of subnetworks is a question that needs further investigation. GLV equations, however, could be interpreted as a special case of Wilson-Cowan equations, wherein the population response function (*S*(*x*) in their notation^10^), which is typically assumed to be a sigmoid function, is a linear function of the overall input (the overall excitation level that is fed into the network). Approximating this response function with a linear function may be valid only if the network is operating in a low firing rate asynchronous irregular regime.

There are, however, subtle differences between GLV equations that describe ecosystems, and the GLV we obtained to approximate the spiking network dynamics. In a two-prey-one-predator system, the coefficient of a prey population variable which affects the population growth of its own or any other prey, is usually taken negative^49^. This usually represents the competition between preys for limited resources. In the EEI network that we considered in this study, there is no direct competition between excitatory neurons for resources, which in this case is represented by the external input. Therefore, the influence of an excitatory population on its own activity or on the other excitatory population’s activity is positive. As it turns out, for negative self-couplings of excitatory populations, and negative couplings between the two excitatory population, there exists a region in the parameter space where *p*_1,1,1_ is a global attractor. This would result in a stable network state, where all subnetworks are active.

For both sample configurations (EEI and IIE) considered in our paper, in the bifurcation diagram obtained from network simulations, there is a gap between the bifurcating regions that should disappear at *a*, *b* = 1. Our simulations indicate that with increasing the network sizes, these small discrepancies vanish. Essentially, the larger the population size, the more precise the GLV system becomes as a low-dimensional description. According to^17,50^, sharp transitions in bifurcation diagrams can happen only in the limit of very large networks. Due to the finite size of our simulated networks, transitions between different states in our diagrams are smooth. Although relative population sizes were taken into account in our model, finite size effects and the exact scaling relations between the number of neurons in each subnetwork and the coefficients in the GLV equation are beyond the scope of this paper.

To summarize, Generalized Lotka-Volterra equations represent an interesting family of systems that can represent a wide dynamical repertoire, such as oscillations, sequential activities, and chaos. Therefore, they can be regarded as a good candidate to model dynamic interactions in neuronal networks on the population level. In this study, we did not aim at identifying the correct time scale of the dynamics from the network parameters, as its dependence is subtle. However, on a qualitative level, we could show that different strengths of couplings between subnetworks can lead to different particular behaviors, and we were able to validate these scenarios by neural network simulations. In fact, in biological systems like neuronal networks, the issue of time scales is under debate^51,52^. As our model is an abstract low-dimensional and simplified representation of a complex biological reality, we cannot claim to represent each and every phenomenon in our model.

The bifurcation analysis in this paper paves the way for deterministic analysis of coupled networks in higher dimensions. It also could be used to study stochastic nonlinear dynamics of such systems, in different regimes of the network. However, in such cases, noise amplitudes can also play role as a bifurcation parameter.

GLV equations have been suggested as a framework to generate metastable systems with saddle points^53,54^ that can exhibit winnerless competition dynamics. This framework allows for the emergence of robust transient dynamics^55^, which was hypothesized to underlie sensory encoding, for example in the olfactory system^33^. In fact, there is evidence that different odor stimuli trigger different transient trajectories and succession of states in a high dimensional neuronal response^35,56,57^. Moreover, Generalized Lotka-Volterra equations have been implicated as models of various cognitive processes, such as decision-making, and sequential working memory^58^. As a model for controlling the desynchronized phase of the sleep cycle, Lotka-Volterra equations have been suggested to replicate the dynamics of excitatory and inhibitory populations^59^. Also, to model the perception of color, such equations were applied to represent the dynamics of competing cortical neurons, which have wave-length dependent activity for redness, greenness, and short wave-length redness, to implement a winner-take-all representation of the phenomenon^60^. Justifying these equations for the collective behavior of spiking networks is an important step towards approaching a mathematical model for brain dynamics, bridging the gap between the different scales of analysis from small neuronal populations to global brain dynamics with a direct link to higher cognitive processes. This eventually will bring us closer to elucidating some fundamental principles of the brain’s complex operations.

## Data Availability

All relevant data are within the paper.
